# Immobilized Forms of the *Ophiostoma piceae* Lipase for Green Synthesis of Biodiesel. Comparison with Eversa Transform 2.0 and Cal A

**DOI:** 10.3390/jof7100822

**Published:** 2021-09-30

**Authors:** María Molina-Gutiérrez, Lorena Alcaraz, Félix A. López, Leonor Rodríguez-Sánchez, María Jesús Martínez, Alicia Prieto

**Affiliations:** 1 Centro de Investigaciones Biológicas Margarita Salas (CIB-Margarita Salas), Consejo Superior de Investigaciones Científicas (CSIC), Ramiro de Maeztu 9, 28040 Madrid, Spain; mariamg.bio@gmail.com (M.M.-G.); leonor@cib.csic.es (L.R.-S.); mjmartinez@cib.csic.es (M.J.M.); 2 Centro Nacional de Investigaciones Metalúrgicas (CENIM), Consejo Superior de Investigaciones Científicas (CSIC), Av. Gregorio del Amo 8, 28040 Madrid, Spain; alcaraz@cenim.csic.es (L.A.); f.lopez@csic.es (F.A.L.)

**Keywords:** biocatalysis, transesterification, recycled oil, FAMEs, sustainability

## Abstract

In this work, we analyzed the suitability of a versatile recombinant lipase, secreted by *Ophiostoma piceae* (OPEr) and produced in *Pichia* *pastoris*, as a catalyst of the synthesis of biodiesel. The enzyme was immobilized by five covalent procedures and by hydrophobicity on functionalized nanoparticles of magnetite or of a novel Zn/Mn oxide named G1. Then, they were tested for green production of biodiesel by solventless enzymatic transesterification of discarded cooking oil and methanol (1:4) at 25 °C. The results were compared with those shown by free OPEr and the commercial lipases Eversa^®^ and Cal A^®^. Several preparations with immobilized OPEr produced high synthesis yields (>90% transesterification), comparable to those obtained with Eversa^®^, the commercial enzyme designed for this application. Three of the biocatalysts maintained their catalytic efficiency for nine cycles. The process catalyzed by AMNP-CH-OPEr was scaled from 500 µL to 25 mL (50 times), improving its efficiency.

## 1. Introduction

Biodiesel is a non-toxic and biodegradable liquid fuel composed of long chain fatty acid alkyl esters with short chain alcohols, generally methanol and ethanol [1]. The synthesis of these compounds can be driven by chemical or enzymatic catalysts, and industrial production is usually done by chemical transesterification of triglycerides, generally with alkaline catalysts [2]. However, in this reaction, soaps are produced, especially if the oil used contains free fatty acids, and this secondary reaction reduces biodiesel yields and makes the separation and purification of the products notably difficult [3,4]. On the contrary, the application of enzymatic methods also allows to synthesize esters from free fatty acids by direct esterification, increasing the total yields. Biodiesel production by lipase-catalyzed transesterification of sunflower oil and methanol was first reported three decades ago [5]. Now, there is a tendency to use non-edible oils or urban wastes as sources of triglycerides, in order to fit within a circular economy scheme and to reduce the expense attributable to raw materials [6], which should not exceed 50% of production costs [1]. Similarly, solvent-free enzymatic bioprocesses are desirable both for environmental and economic reasons. Although the presence of a reaction solvent increases the solubility of oils and alcohol, reduces viscosity, and improves mass transfer [7], solventless reactions are neat, facilitate easy separation, workup and purification, and minimize the production of waste [8].

Water-soluble lipases are homogeneous catalysts widely used for enzymatic transesterification, but its application at large-scale is hampered by the high cost of the biocatalyst, its low reusability, the need to be separated from the reaction products, and the limited stability of many enzymes in the presence of methanol [9,10]. However, the development of heterogeneous biocatalysts in which the enzyme is made insoluble through its attachment to a solid support can help to solve some of these problems. A proper immobilization procedure may improve enzyme stability or specificity, reduce the possibility of being inhibited, provide operational flexibility, and eliminate downstream operations allowing a simple separation from the product, enabling its recovery and reuse, thus reducing operational costs [9,10,11,12,13,14,15]. The carriers and techniques used for immobilization are varied [10,16] and it is important to keep in mind that the properties of the final biocatalyst rely on the interaction between the carrier, the enzyme, and the method for protein attachment [17]. The techniques used for lipase immobilization include adsorption, covalent binding, entrapment, encapsulation, and cross-linking [6,18], and there are not general rules to determine which are the optimal carrier and immobilization strategy for a determined enzyme and reaction [12]. In general, the protein structure suffers changes during immobilization, thus it interesting to test several carriers and strategies to select the most efficient for the target application [19]. Among the materials that can serve as immobilization carriers [20], inorganic nanoparticles are attracting growing interest for their excellent behavior [21] and because particle size can be tailored attaining homogeneous and well-defined core-shell nanoparticles with a thick enzyme shell [22]. Among these, magnetic nanoparticles (MNPs) are very appreciated due to their high surface area, the availability of many hydroxyl groups that are easily modified facilitating a strong (covalent) binding of the enzyme, their high mechanical stability and low porosity that minimizes steric hindrances, and the easy separation of the biocatalyst from reaction mixtures by using an external magnetic field [21]. Several lipases immobilized on silica-coated MNPs have proved to be versatile biocatalysts for biodiesel production [9].

Adsorption is probably the most frequent immobilization method for these enzymes. True lipolytic lipases experience the phenomenon of interfacial activation, that involves the transition from the inactive (closed) form of lipases to their active (open) configuration at an oil–water interface, leaving two highly hydrophobic regions exposed to the environment [23]. Thus, in the presence of hydrophobic materials like some immobilization carriers, this mechanism mediates the quick and simple adsorption of the protein [12,24,25]. Triglyceride lipases are particularly sensitive to this phenomenon [23] and they are usually hyperactivated when immobilized on hydrophobic carriers which, in addition, is accompanied by enzyme stabilization and purification [25,26]. In addition, the use of a hydrophobic carrier avoids the adsorption of water and glycerol, which produces diffusional limitations for hydrophobic substrates [4,14,27]. Another mechanism used for specific adsorption of a protein to a carrier is ionic binding, in which the charged groups of the carrier interact with charged amino acid residues on the enzymes in the immobilization conditions, for example, the ε-amino groups of lysins or the carboxyl groups of glutamic and aspartic acids [28].

Nevertheless, covalent immobilization has the advantage of irreversible binding of the protein to the support, avoiding leaching during the catalytic process [10,18,28]. The existing protocols for covalent attachment are numerous and generally involve the side chains of different amino acids located in the protein surface which are not essential for its catalytic activity [22] and various active groups in the supports [28]. Amino-functionalized carriers are widely used to this purpose, and they can be further activated with bifunctional reagents such as glutaraldehyde (GA), grafting reactive aldehyde groups in the carrier’s surface and allowing multipoint attachment of the protein [25,29]. This versatile reagent is also generally used as cross linker for covalent immobilization as CLEAs (cross-linked enzyme aggregates) or mCLEAs (magnetic CLEAs, obtained including magnetic nanoparticles in the immobilization mixture) [30]. However, in the case of glycoproteins, the binding to the support can also be carried out through aldehyde groups obtained by soft chemical oxidation of the carbohydrate chain, and not through the peptide chain [28]. This procedure has been scarcely used, although it leads to oriented enzyme immobilization on the carrier surface and high retention of lipase activity, compared with the same carrier activated with GA [28,31].

**Figure 1:**
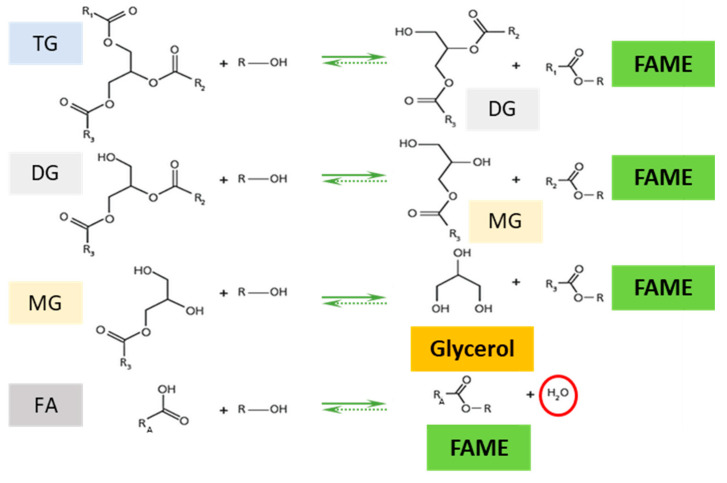
Simplified schemes of the transesterification of a triglyceride and of direct esterification of a free fatty acid with methanol to synthesize biodiesel. TG: Triglyceride; DG: Diglyceride; MG: Monoglyceride; FAME: Fatty acid methyl ester.

Figure 1 schematizes the enzymatic transesterification of triglycerides in an organic medium (some water can be included in the reaction mixture) containing free fatty acids and a short-chain alcohol. In these conditions, the reaction is biased to synthesis. The reaction is complex, since intermediate reaction products (diglycerides, monoglycerides) are released during transesterification, that occurs sequentially. To complete the reaction, three molecules of alcohol are required per triglyceride molecule, producing three molecules of esters and one of glycerol. Free fatty acids are also transformed into the corresponding esters by direct esterification, releasing a water molecule.

Differently to enzymatic esterification, which is thermodynamically controlled, transesterification is kinetically driven and the maximum conversion yields result from several kinetic properties of the enzyme [32,33,34]. In the presence of water, the reaction can revert to hydrolysis, lowering product yields. The generally accepted mechanism of action for lipases is of the Ping-Pong Bi-Bi type, although several different models have been described to explain the transesterification of glycerides. The one-step model describes the direct alcoholysis of triglycerides, in which the alcohol is responsible for the breakdown and esterification of the released fatty acid. The two-step model requires the presence of some water: in a first phase, water hydrolyzes the triglyceride, and in the second stage, the fatty acids released are esterified. Canet et al. [35] reviewed the mechanisms described and proposed a new one that takes into account the presence of free fatty acids, confirming that both processes coexist. Andrade et al. [36] corroborated that the process involves hydrolysis and synthesis reactions, with predominance of synthesis. Canet et al. [35] also showed that polar molecules in the reaction medium, like free fatty acids, mono- and diacylglycerols, protect the enzyme by buffering the high polarity of methanol, improving the reaction rate and the stability of the biocatalyst.

Many free and immobilized microbial lipases have been tested as catalysts of the synthesis of biodiesel [9]. In the last years, enzymes like *Candida antarctica* lipase A (Cal A) [37] and very specially Eversa Transform^®^, a genetically-modified variant of the lipase from *Thermomyces lanuginosus* designed by Novozymes for the synthesis of biodiesel, have been used in this application [36,38,39,40,41,42]. Both biocatalysts have been immobilized by hydrophobicity on octyl agarose beads [24,39,43], Cal A was also covalently attached to chitosan-coated magnetic nanoparticles activated with GA [44], and mCLEAs of Eversa Transform^®^ were prepared and tested to produce biodiesel [41]. The recombinant versatile lipase from *Ophiostoma piceae* (OPEr) has shown to be a robust biocatalyst, whose activity is maintained or improved upon immobilization. The activity and efficiency of various preparations with immobilized OPEr have been previously tested in two reactions of industrial interest: the synthesis of flavors by direct esterification (in biphasic systems) [45], and the production of sterol esters by transesterification in a solvent-free medium [31]. In this work, we will present the results of biodiesel production from methanol and cooking oil wastes by a green procedure. Six variants with immobilized OPEr are evaluated as catalysts of the reaction, performed at room temperature (25 °C) without adding water or cosolvent. The enzyme is covalently immobilized on nanoparticles of magnetite bearing superficial amino or aldehyde groups, as well as non-covalently (by hydrophobic interaction) on a magnetic support with octyl groups in the surface. A novel Zn/Mn oxide without magnetic properties is also tested as an immobilization scaffold after being functionalized with amino or aldehyde groups. The results are compared with those shown by the free OPEr and the commercial lipases Eversa^®^ and Cal A^®^.

## 2. Materials and Methods

### 2.1. Enzymes

The recombinant lipase from *O. piceae* was produced in *Pichia pastoris* as previously reported [46]. The supernatant of 4-day-old cultures was filtered and concentrated by ultrafiltration using an YM3 Amicon device with a 50 kDa membrane for obtaining the OPEr crude enzyme. The commercial lipases Cal A and Eversa^®^ Transform 2.0 were a gift from Novozymes.

### 2.2. Evaluation of Protein and Enzyme Activity

The amount of protein was assessed by measuring absorbance at 280 nm (UV) using a NanoDrop 2000 (Thermo Fisher Scientific, Waltham, MA, USA). The activity of the catalyst was determined monitoring at 410 nm the release of *p*-nitrophenol from hydrolysis of 1.5 mM *p*-nitrophenyl butyrate (*p*NPB, Sigma-Aldrich, St. Louis, MO, USA) in 20 mM Tris−HCl pH 7.0 at room temperature using a Shimadzu UV-160A spectrophotometer. One unit of activity (1 U) is defined as the amount of enzyme used to release 1 μmol of *p*-nitrophenol (ε_410_ = 15,200 M^−1^ cm^−1^) per minute under the defined conditions.

### 2.3. Immobilization of Lipases and Determination of the Lipase Activity

The concentrated enzyme crude containing the recombinant lipase from *O. piceae* was immobilized by several procedures. The SiMAG-octyl scaffold used for non-covalent (hydrophobic) immobilization bears an octyl (C8) surface group and was provided by Chemicell (Berlin, Germany). All other functionalized supports were prepared in the laboratory using commercial Fe_3_O_4_ magnetic nanoparticles (MNPs) or a new type of non-magnetic nanoparticles, whose formula is Zn_0.25_Mn_2.75_O_4_ (G1). This carrier was synthesized using material recovered from discarded alkaline batteries, and its preparation and purification have been described elsewhere [47]. The magnetic nanoparticles were characterized by X-ray diffraction, scanning and transmission electron microscopy and Fourier transform infrared spectroscopy under conditions previously reported [45], using the same methods to characterize the G1 non-magnetic carrier. All scaffolds were coated with a thin layer of Si and amino groups were incorporated on their surface (AMNP and AG1 carriers) by treatment with 99% (3-aminopropyl)triethoxysilane (APTS, Sigma-Aldrich), followed or not by activation with glutaraldehyde [45]. If activated, the carriers are denominated as AMNP-GA and AG1-GA. The crudes with OPEr were immobilized by four procedures: (i) For immobilization by adsorption, the protein solution was carefully mixed with SiMAG-octyl nanoparticles in 100 mM Tris-HCl buffer pH 7 [45] to produce the catalyst SiMAG-octyl-OPEr; (ii) covalent attachment to AMNP-GA or AG1-GA was attained by mixing the protein solution with the activated carrier in 100 mM Tris-HCl buffer solution, pH 7 [45]. These catalysts were denominated AMNP-GA-OPEr and AG1-GA-OPEr; (iii) magnetic cross-linked enzyme aggregates (mCLEAs-OPEr) were prepared in the presence of the activated carrier AMNP-GA by precipitation with ammonium sulfate and crosslinking with GA in 100 mM Tris-HCl buffer solution, pH 7 [45]; (iv) for immobilization through the carbohydrate chains, the protein solutions were first subjected to a soft oxidation with NaIO_4_ to produce aldehyde groups from vicinal diols in the sugar chain. Then, the solution was allowed to react with AMNPs or AG1 under reducing conditions, stabilizing the covalent linkage with NaBH_4_ [31]. These catalysts were denominated AMNP-CH-OPEr and AG1-CH-OPEr.

The details on functionalization, protein immobilization, and characterization are given in Molina-Gutiérrez et al. [31,45]. In all cases, the immobilization yield (%) was calculated from the difference between offered activity and residual activity in the supernatant at the end of the immobilization period. The activity recovery was determined by considering the OPEr activity initially offered for immobilization and the activity of the immobilized catalyst. Both the residual activity in the supernatants after immobilization and the specific activity of the immobilized catalysts (mU/mg carrier) were determined as explained in Section 2.2. The optimal amount of protein offered for immobilization (per mg of carrier: 1050 mU for SiMAG-octyl, 1500 mU for AMNP and 750 mU for AMNP-GA) was selected in preliminary tests as a compromise between immobilization yield and specific activity of the insoluble biocatalysts. For comparison, the commercial preparations of Eversa and Cal A were immobilized in one of the conditions tested for OPEr.

One of the immobilized forms of OPEr, AMNP-CH-OPEr, was produced at larger scale (10×) using 200 mg of OPEr (previously treated to generate aldehydes in its carbohydrate chains) and 10 g of AMNPs, allowing it to react in the same conditions described by Molina-Gutiérrez et al. [31].

### 2.4. Synthesis of Biodiesel and Operational Stability of the Immobilized Enzymes

The production of fatty acid methyl esters (FAMEs) was routinely evaluated in solvent-free reactions containing 500 μL of cooking oil wastes, methanol (1:4 molar ratio), and 100 mg of the corresponding immobilized catalyst. Gas-chromatography analysis of the oil allowed the identification of triolein and other triglycerides as major components, although low amounts of the diglycerides 1,3-dioleoyl glycerol and 1-oleoyl-3-palmitoyl glycerol were also detected. The peak of free oleic acid was nearly undetectable, suggesting that the acid value of the oil was very low. Fatty acid composition was determined after chemical methanolysis of the oil sample (75% methyl oleate, 15% methyl palmitate and 9% methyl stearate). The vials were kept at 25 °C with rotational agitation at 100 rpm in a mixer for 24 h. Methanol was added in three steps (0 h, 5 h, and 9 h). To evaluate the recyclability, the nanobiocatalysts were separated after each reaction cycle using an external magnet and washed with isooctane and 20 mM Tris-HCl, pH 7. The time course of the synthesis of biodiesel was monitored by gas chromatography, determining the production of methyl esters and the consumption of triglycerides at different times. The reactions were performed in duplicate. To assess the scalability of this approach, methanolysis reactions were performed with 5 g of AMNP-CH-OPEr in a final volume of 25 mL under the same reaction conditions.

### 2.5. Chromatographic Methods for Sample Analysis

The discarded domestic oil used as substrate and treated samples of 5, 9 and 24 h were analyzed by gas chromatography/mass spectrometry (GC/MS) in an Agilent instrument 7890A/5975C using a 30 m DB5-HT column and a temperature program of 180 to 205 °C (ramp rate 3 °C/min), then to 220 °C (10 °C min), and finally from 220 to 400 °C, maintaining the final temperature for 5 min. The compounds detected were identified using commercial standards analyzed under identical conditions, or through their mass spectra using the NIST 2011 library and the ChemStation software (Agilent, Palo Alto, CA, USA). The time course of the reactions was routinely monitored by gas chromatography (GC) in an Agilent 7890A with FID detection and a 5-m SPB-1 column (Supelco, Bellefonte, PA, USA). Aliquots of 15 µL, containing 60 mM cholest-3-one as internal standard, were taken at 0, 5, 9 and 24 h, diluted with isooctane to a final volume of 300 µL, and analyzed by GC using a multistep temperature program from 140 to 350 °C.

## 3. Results

### 3.1. Characterization of the Novel Zn/Mn Mixed Oxide Used as Immobilization Carrier

**Figure 2:**
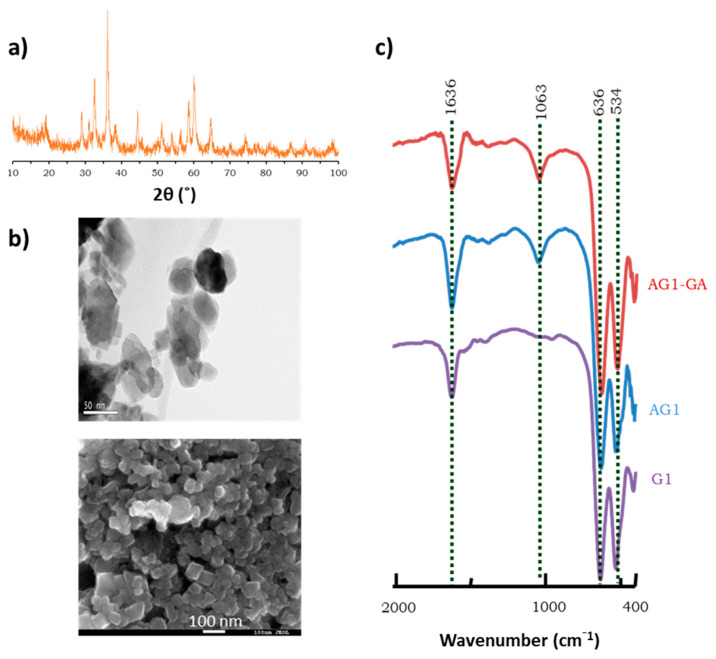
Characterization of the G1 carrier. (**a**) X-ray diffraction (XRD) pattern of G1. (**b**) TEM (upper) and SEM (lower) micrographs of G1. (**c**) FTIR spectra of G1, AG1, and AG1-GA.

The nude, amino-functionalized, and GA-activated magnetic carriers have been previously characterized using Fourier transform infrared spectroscopy (FTIR) and by transmission (TEM) and scanning electron microscopy (SEM), confirming also that the magnetic properties of the carrier were maintained after immobilization [31,45]. Figure 2 displays the data of the different techniques used for characterization of the novel G1 non-magnetic carrier.

G1 is a mixed oxide of Mn/Zn of high purity (95–96%). Its RXD pattern (Figure 2a) shows signals attributable to a tetragonal symmetry and a spinel-like structure, in accordance with the JCPDS database (No. 24-1133), with stoichiometry Zn_0.25_Mn_2.75_O_4_. The observation of the G1 nanoparticles by TEM and SEM (Figure 2b,c) showed that, unlike the MNPs, their shape and size are not homogeneous, so it is impossible to give a value of their average diameter. In general, its size (>50 nm) is larger than that of magnetic supports. The heterogeneity of these materials is expectable, since they were synthetized from a waste by a new, non-industrial process, without using any matrix for size or shape.

In FTIR spectra (Figure 2c), the band at 534 cm^−1^ is attributed to Mn-O vibrations, and the band around 636 cm^−1^ is related to spinel ZnMn_2_O_4_ [48]. In the functionalized supports, the band at 1063 cm^−1^ corresponds to the SiO-H groups. The band at 1647 cm^−1^ is intensified in the functionalized samples due to the N-H stretching vibrations of the free amino groups [49]. After silanization and functionalization with the APTS reagent, the average amount of amino groups incorporated to the G1 nanoparticles was 10.8 ± 3.4 μmol/g of support, a value comparable to that determined for AMNPs [45].

### 3.2. Immobilization of OPEr

Four procedures that involve different regions of the protein were tested for OPEr immobilization. The covalent preparations were obtained by attaching the protein to two GA-activated supports, by preparation of mCLEAs with the AMNP-GA carrier, and by binding the (glyco)protein to the amino groups of the two aminated supports through aldehydes chemically generated in the carbohydrate (CH) chains of the enzyme (AMNP-CH and AG1-CH preparations). This is the only case in which the protein portion does not participate directly in binding [50]. In addition, OPEr was also adsorbed on the hydrophobic SiMAG-octyl carrier.

Every preparation with immobilized OPEr will be considered as a different biocatalyst, since the protein is attached in different ways depending on the carriers’ composition, surface functional groups, and immobilization conditions, which influence enzymatic activity. The immobilization parameters determined for the six heterogeneous biocatalysts prepared with OPEr are summarized in Table 1. The six nanobiocatalysts produced are active against *p*NPB, although the specific activity of those with the carrier activated with glutaraldehyde was around two to three times lower than the others, since the protein load accepted by this support was also much lower.

The immobilization yield describes the percentage of total enzyme activity from the free enzyme solution that is immobilized, and it is determined by measuring the residual activity that remains in solution after immobilization, which is subtracted from the starting activity. The success of immobilization is given by two parameters: the immobilization efficiency, that describes the percentage of bound enzyme activity that is observed in the immobilizate, and the recovered (or expressed) activity that compares the activity of the immobilizate to that of the total starting activity of the free enzyme [15].

According to the results in Table 1, the most successful immobilization methods were non-covalent deposition (SiMAG-octyl-OPEr) and mCLEAs. In both cases, the enzyme was immobilized in high yield, and the efficiency and recovered activity were also over 80%. Their specific activity was in the range of those measured for the two catalysts in which OPEr was attached through the carbohydrate chains (CH). However, the immobilization process was less efficient in the last, as deduced from their intermediate values of recovered activity and efficiency. Finally, immobilization of OPEr to GA-activated nanoparticles yielded the worst results, with low values for all the determined parameters. Nevertheless, the high immobilization efficiency value calculated for the AG1-GA-OPEr preparation suggests an hyperactivation of the immobilized enzyme.

### 3.3. The Six Nanobiocatalysts Catalyze the Synthesis of Biodiesel

Once the immobilization was completed and the activity of the nanobiocatalysts on *p*NPB verified, we tested their activity and efficiency in the synthesis of methyl esters (FAMEs) from cooking oil wastes. To compensate for possible evaporation of the alcohol in the reaction and during sampling, it was chosen to work with a methanol:oil molar ratio of 4:1, developing the reactions under green conditions: Solvent-free, 25 °C, and without adding molecular sieves or drying agents.

**Figure 3:**
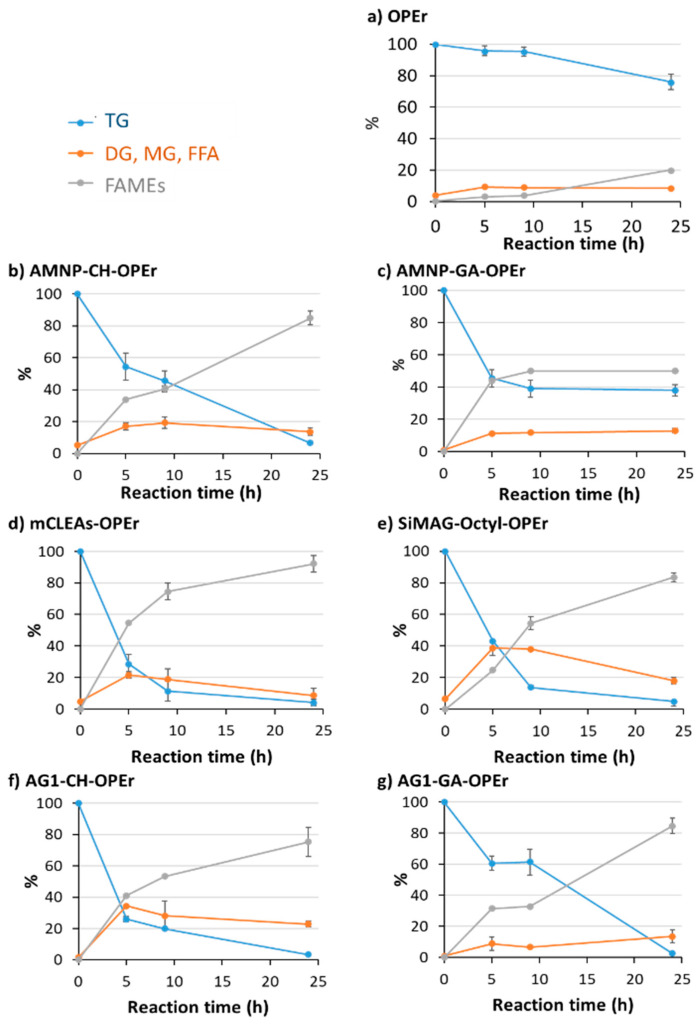
Time course of the enzymatic transesterification of recycled cooking oil and methanol catalyzed by OPEr and its immobilized biocatalysts, showing the percentage of triglycerides, FAMEs, and other reaction products. (**a**) Soluble OPEr; (**b**) AMNP-CH-OPEr; (**c**) AMNP-GA-OPEr; (**d**) mCLEAs-OPEr; (**e**) SiMAG-octyl-OPEr; (**f**) AG1-CH-OPEr; (**g**) AG1-GA-OPEr. The reactions were developed over 24 h at 25 °C, with a rotational stirring (100 rpm), an oil:methanol ratio of 1:4 and 100 mg of each nanobiocatalyst. TGs: Triglycerides; DGs: Diglycerides; MGs: Monoglycerides; FFAs: Fatty acids; FAMEs: Methyl esters.

The time course of the reactions is shown in Figure 3. It can be observed that the immobilization of OPEr drastically increased its ability to synthetize biodiesel. The soluble enzyme (Figure 3a) produced 20% FAMEs in 24 h, while the reactions with immobilized enzyme yielded between 50–90% (Figure 3b–g), depending on the carrier and immobilization conditions. At this point, it is necessary to consider that, for technical reasons, these reactions contained 100 mg of each nanobiocatalyst, so the enzymatic dose is not uniform since the preparations had different specific activity (Table 1). This is especially relevant for AMNP-GA-OPEr, whose specific activity was between two and three times lower than that of the rest, justifying its reduced synthetic activity. The efficiency of this catalyst is likely to be comparable to that of the others if the catalytic dose were leveled. Leaving this preparation aside and considering the value of the calculated standard deviations, the results indicate that the activity of all nanobiocatalysts in the synthesis of FAMEs is similar.

Table 2 shows the results of the chromatographic analysis of 24 h reactions, showing the percentage of acyl-glycerides, free fatty acids, and methyl esters. The free enzyme and the variant immobilized on the AMNP-GA carrier kept high amounts of triglycerides, AG1-GA-OPEr accumulated some diglycerides, and the non-covalent preparation SiMAG-octyl-OPEr showed to have nearly 15% free fatty acids at the final reaction time.

### 3.4. Recyclability of OPEr Nanobiocatalysts in Biodiesel Synthesis

**Figure 4:**
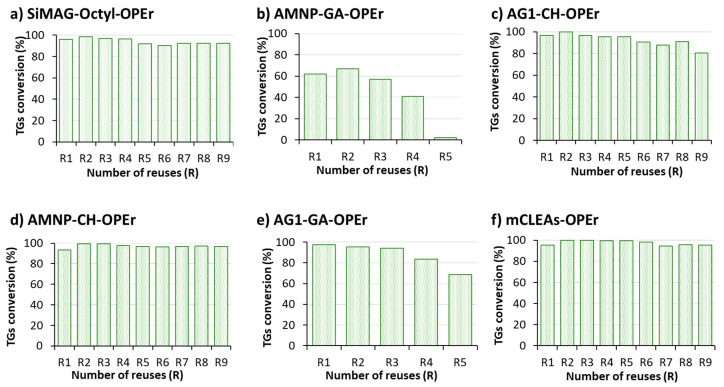
Operational stability of OPEr nanobiocatalysts in nine consecutive 24 h transesterification cycles of cooking oil wastes and methanol. (**a**) SiMAG-octyl-OPEr; (**b**) AMNP-GA-OPEr; (**c**) AG1-CH-OPEr; (**d**) AMNP-CH-OPEr; (**e**) AG1-GA-OPEr; (**f**) mCLEAs-OPEr. The reactions were developed at 25 °C, with a rotational stirring (100 rpm), an oil:methanol ratio of 1:4, and 100 mg of each nanobiocatalyst.

To check their operational stability, the six OPEr nanobiocatalysts were subjected to up to nine successive reaction cycles. In cases where a marked decrease in activity was observed in the fifth consecutive reaction, recycling was stopped. Figure 4 presents the percentage of triglycerides converted in each reaction cycle. It is interesting to note that, in the second reaction cycle (R2), all OPEr preparations, apart from AG1-GA-OPEr, slightly increased the biodiesel production yield. Except the variants immobilized on carriers activated with GA (regardless of if the naked material was magnetite or the Zn/Mn oxide), that lost more or less catalytic activity in this reaction after several reuses, the other nanobiocatalysts maintained almost 100% of their conversion activity after nine reaction cycles. The only preparation that experienced a clear loss of activity is AMNP-GA-OPEr (Figure 4b), which stopped working in the fifth cycle (R5), and AG1-GA-OPEr maintained 70% residual activity in R5 (Figure 4e). Immobilization by non-covalent hydrophobic interaction (Figure 4a), as well as mCLEAs (Figure 4f) and the covalent preparations that used the AMNP and AG1 carriers and the aldehydes generated in the sugar chains of the proteins (Figure 4c,d), preserved best the catalytic activity of OPEr in this reaction, although the AMNP-CH-OPEr biocatalyst seemed to be the most stable.

### 3.5. Biodiesel Synthesis Catalyzed by Commercial Enzymes

The soluble commercial lipases Cal A^®^ and Eversa^®^ were tested under the same alcoholisis conditions described in the previous section. Both enzymes were also immobilized as AMNP-CH-, since this procedure gave good immobilization yields, specific activity, and operational stability for OPEr and does not involve the peptide chain in binding.

**Figure 5:**
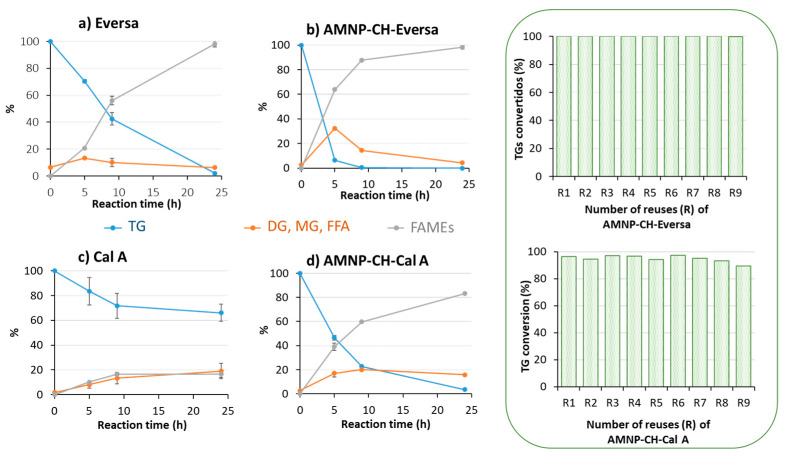
Enzymatic transesterification of discarded cooking oil and methanol catalyzed by: (**a**) soluble Eversa; (**b**) AMNP-CH-Eversa; (**c**) soluble Cal A; and (**d**) AMNP-CH-Cal A. The bar plots framed in green represent the operational stability of the immobilized enzymes in nine consecutive 24 h transesterification cycles. The reactions were developed over 24 h at 25 °C, with a rotational stirring (100 rpm), an oil:methanol ratio of 1:4, and 100 mg of each nanobiocatalyst. TGs: Triglycerides; DGs: Diglycerides; MGs: Monoglycerides; FFAs: Fatty acids; FAMEs: Methyl esters.

Eversa^®^ is the commercial enzyme designed by Novozymes for the synthesis of biodiesel. As such, it showed excellent behavior under these reaction conditions, both in its soluble and immobilized forms, converting the entire substrate into FAMEs (Figure 5a,b). However, the free enzyme produces around 60% of FAMEs after 9 h of reaction, while the immobilized form synthesized around 80% of biodiesel in the same period.

The tests carried out with the soluble form of Cal A^®^ demonstrated its low efficiency in biodiesel synthesis, like that observed for OPEr (Figure 5c and Figure 3a, respectively). However, AMNP-CH-Cal A produced 83% FAMEs and hydrolyzed virtually all available TGs (Figure 5d). When analyzing the reaction mixture, 13% of free FAs were detected (data not shown).

Concerning operational stability, both AMNP-CH-Eversa^®^ and AMNP-CH-Cal A maintained their activity over nine reaction cycles, confirming their robustness (Figure 5), and the excellent recyclability of the AMNP-CH preparations.

### 3.6. Reaction Scalability

**Figure 6:**
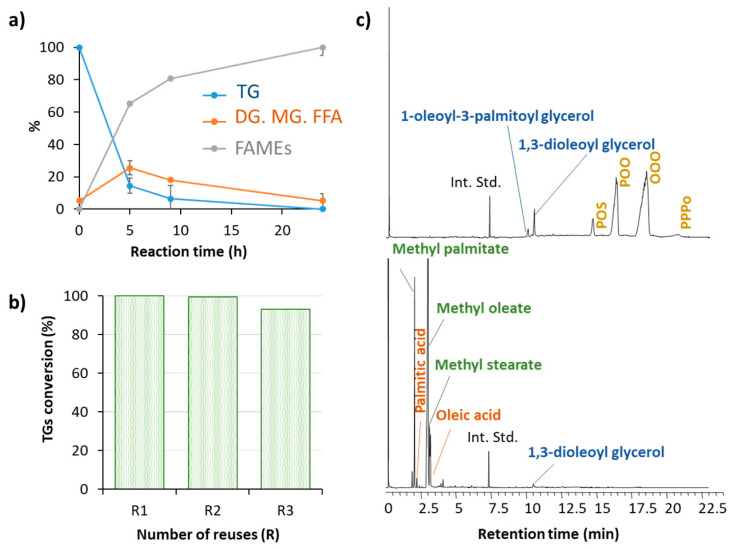
(**a**) Enzymatic transesterification of 25 mL of discarded cooking oil and methanol (25 °C, 100 rpm, oil:methanol 1:4, 5 g AMNP-CH-OPEr); (**b**) recyclability of AMNP-CH-OPEr in reactions of 24 h; (**c**) chromatograms of the reaction mixture at 0 h (upper graph) and 24 h (lower graph). OOO: Triolein; OOP: Palmitodiolein; POS: 1-palmitoyl, 2-oleyl, 3-stearoyl glycerol; PPPo: Palmitoleyldipalmitin.

All the above reactions were performed in a final volume of 500 μL, which is a manageable size to test the efficiency and general properties of the battery of nanobiocatalysts. Still at the laboratory level, we prepared AMNP-CH-OPEr in greater quantity and repeated the methanolysis process on a larger scale, to check if the good catalytic behavior and the simple handling of the reaction were maintained throughout the process. Although no lipase activity was detected in the supernatant after immobilization, the specific activity and recovery of activity determined for this batch were somewhat lower than those of a reaction with 1 g of AMNPs and 20 mg of protein, developed in parallel. The alcoholysis was successfully completed (Figure 6a,c), and the conversion of triglycerides to methyl esters was nearly full. This reaction was repeated three consecutive times, with little loss of activity (Figure 6b).

## 4. Discussion

### 4.1. Immobilization of OPEr. Protein-Scaffold Interactions and Assessment of Enzyme Activity upon Immobilization

Immobilization of a protein involves its attachment to an insoluble solid support. If the activity of the enzyme resists this process, the immobilized biocatalyst is often more stable than the free enzyme, can be easily separated from the reaction mixture facilitating the purification of the products, and can be reused [15,16,28,51,52,53,54]. However, it is not easy to predict which of the multiple immobilization carriers and methods will be best suited to produce a robust biocatalyst for a given application, and the usual approach is that of trial and error. Here we have tested two types of inorganic nanoparticles as basal scaffolds: magnetite and a novel Zn/Mn oxide produced from recycled batteries denominated G1 and used as immobilization carrier for the first time.

**Figure 7:**
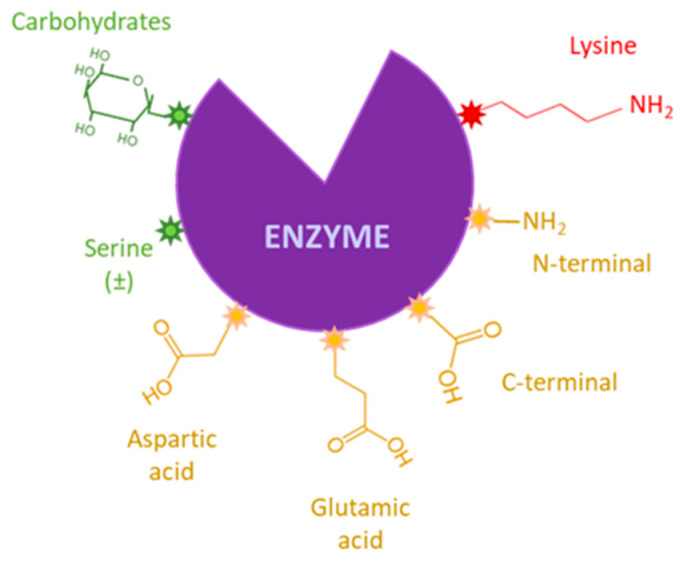
Scheme of amino acids that can serve as anchors in immobilization. Red: Used very frequently; yellow: Frequently used; green: Not frequently used. Adapted from [28].

Most immobilization methods involve the interaction of specific areas of the peptide chain with the reactive groups placed in the scaffold’s surface. Thus, depending on the reactivity of these groups, different regions of the peptide chain will be involved in the attachment. However, only few amino acids can mediate this binding thanks to their reactivity (Figure 7), and the participation of one or the other will depend on the scaffold composition, the functional groups placed on its surface, and the conditions in which the immobilization is carried out [28].

OPEr was covalently attached to the insoluble scaffolds using several strategies that involve different structural regions. First, the synthesis of mCLEAs is probably the least specific of the methods used, since proteins are massively precipitated and crosslinked. However, the procedure used here involves a first (probably ionic) interaction of the protein with the AMNP-GA carrier, which conditions the activity of the preparation, since mCLEAs of OPEr synthesized by direct addition of precipitant and crosslinker were inactive in this reaction (data not shown). Second, the immobilization of OPEr by attachment by its oxidized glycidic chains to the AMNP carrier is an unusual method that, as already mentioned, may better preserve enzyme activity. The mobility of the peptide chain should still be restricted due to immobilization, although protein structure and activity are probably less affected than in the other procedures. Finally, the neutral pH used for immobilization of OPEr to the AMNP-GA carrier [45] makes it unlikely that the ε-amino groups of surface lysins are activated since their pKa is very high. Under these conditions, it is quite possible that the proteins have joined the activated scaffold mainly by their N-terminal amino acid [29], although we cannot exclude the intervention of other regions with reactive amino groups at neutral pH. Despite the proximity of the N-terminal to the lid region of OPEr [55], it appears that immobilization through this point does not affect its opening, since the immobilized biocatalyst is active. In addition to covalent immobilizations, a commercial magnetic support functionalized with surface octyl groups (SiMAG-octyl^®^) was also used to immobilize OPEr by hydrophobic interaction.

As observed in Table 1, all the catalysts with immobilized OPEr hydrolyzed the *p*NPB used as a model substrate, confirming that they were active. As previously noticed, the specific activity of those with the carrier activated with glutaraldehyde was lower than the others, since the protein load accepted by this support was also much lower. In addition, the data show that the specific activity determined for the enzyme immobilized on AG1 and AG1-GA in this reaction was higher than those calculated for the analogous magnetic supports (AMNP and AMNP-GA). The parameters determined for AG1-GA-OPEr suggest that the protein is hyperactivated when immobilized in these conditions, since the immobilization efficiency is 126% while the immobilization yield (43%) and recovered activity (41%) are low. The reasons that justify this behavior merit further investigation. These results demonstrate that not only the surface groups of the support affect enzyme activity, but also its chemical composition, and confirm the importance of biocatalyst engineering in order to select the most suitable for a given application [15,17,56].

On the contrary, despite hyperactivation of several lipases immobilized by interfacial adsorption on hydrophobic carriers being described a long time ago [57], the parameters calculated for SiMAG-octyl-OPEr (immobilization efficiency—82%; immobilization yield—99%; recovered activity—80%) do not show any hyperactivation of the enzyme, confirming previous findings [58]. According to Derewenda et al., the activation of lipases at an oil–water interface is a unique property which is evolved in response to the problem of handling insoluble substrates, and it distinguishes true lipolytic proteins from esterases which act upon soluble esters [23]. The increase of the lipolytic activity has usually been related to the displacement of the lid [59,60]. However, in spite of having lids that must be opened to allow the access to the active center, hyperactivation has not been observed either in OPEr or in the isoforms CRL3 and CRL4 of *Candida rugosa,* which are structurally similar to OPEr and possess relevant esterase activity [58]. In addition, two of them are dimeric (there is not information on the tertiary structure of CRL4), with their lid participating to a greater or lesser extent in dimer formation [61], which may also help to explain this behavior.

### 4.2. Activity of Free and Immobilized OPEr in Synthesis of Biodiesel. Comparison with Eversa Transform 2.0 and Cal A

In this work, we decided to use environmentally friendly conditions for the enzymatic methanolysis of triglycerides from oil wastes, without consuming energy (25 °C) or unnecessary organic solvents. In the conditions assayed, the soluble lipases OPEr and Cal A displayed low efficiency synthetizing biodiesel. The presence in the reaction medium of around 10% buffer in which the enzyme is dissolved can influence the performance of alcoholisis [18]. However, when the enzyme is immobilized, most water can be removed after dispensing the biocatalyst in the reaction vial. On the contrary, the reactions catalyzed by free Eversa^®^ reached completion regardless of the water content, highlighting the extreme efficiency of this biocatalyst specifically designed for this application by Novozymes. This product, launched on the market in 2014, was advertised as the first enzymatic solution for the synthesis of biodiesel from waste oils [62].

There are many articles published on the use of lipases immobilized on different supports for the synthesis of biodiesel, using or not cosolvents and water capture systems, and under various conditions and reaction times. The amount of biocatalyst used is also highly variable, and for lipases immobilized on AMNPs, these percentages are usually between 17.3–40% (*w*/*w*) [63]. This disparity of conditions makes it difficult to compare the data described in the bibliography with those presented in this work. For this reason, one of the methods was chosen to immobilize the two commercial lipases with the objective of comparing the activity of OPEr in the same reaction conditions. The free form of the lipase Cal A^®^ has been reported to catalyze the synthesis of biodiesel from waste frying palm oil [37]. The maximum FAMEs yield (94.6 ± 1.4%) was achieved under reaction conditions optimized by RSM: 16.6 wt% water content and 5.5 wt% lipase load at 30 °C for 22 h.

The results depicted in Figure 3, Figure 4 and Figure 5 demonstrate that any of the immobilized preparations improved the efficiency of the corresponding free enzymes in this reaction and allowed the recovery of the biocatalyst for further reuse. The robustness and efficiency of OPEr in this reaction improved markedly in five of the OPEr nanobiocatalysts, with synthesis yields between 75–92% (Table 2), and three of them maintained their catalytic efficiency for nine cycles (Figure 4). Concerning the commercial catalysts, this is the first time that either Cal A or Eversa Transform 2.0 are immobilized through the aldehyde groups generated in their sugar chains. In fact, to the best of our knowledge, there are no reports describing the immobilization of lipases by this procedure, except a previous paper from our group in which AMNP-CH-OPEr was successfully applied to synthetize stanol esters [31]. The immobilized AMNP-CH-Eversa seemed to have a faster reaction rate as compared with the free form, and the efficiency of AMNP-CH-Cal A improved considerably with respect to the free enzyme. In the best OPEr catalysts, the yield of FAMEs and the operational stability are slightly lower to those observed for Eversa^®^.

Many publications describe the application of soluble Eversa^®^ in synthesis of biodiesel, but, as it is a low-cost enzyme, the interest in its immobilization is limited. The study of Remonatto et al. [38] evaluated the activity of Eversa immobilized on four hydrophobic supports in alcoholysis of sunflower oil. The authors observed the total conversion of the oil into methyl esters in short times, but the reactions were developed at 40 °C with hexane as co-solvent and in strict anhydrous conditions. Although the enzyme immobilized on Sepabeads-C18 produced esters in a short time, its stability against methanol was very poor, maintaining the activity for three cycles and preserving 75% in the fourth. Therefore, our results with the covalent preparation of Eversa^®^ make a clear difference in terms of the recyclability of this enzyme after immobilization. Other works report the non-covalent immobilization of Eversa in agarose beads functionalized with octyl or aminated groups and their use in hydrolysis reactions [43], and the synthesis of mCLEAs-Eversa to produce biolubricants [40].

The literature describes biodiesel synthesis assays using enzymes immobilized on GA-activated nanoparticles. FAMEs were produced by transesterification of soybean oil with methanol catalyzed by the lipase of *Thermomyces lanuginosus* immobilized on AMNP-GA [64], with a conversion greater than 90% (30 h, 50 °C, methanol:oil 3:1), and Miao et al. [13] obtained 90% biodiesel with Cal B^®^ immobilized in this way.

Several lipases have been immobilized as mCLEAs and applied in the synthesis of biodiesel. Cruz-Izquierdo et al. [63] described the maintenance of the activity of Cal B^®^ mCLEAs in the synthesis of propyl esters, but reducing the reaction time from 72 to 24 h, at the cost of a lower yield. Similarly, the lipases of *Aspergillus oryzae* ST11, *Burkholderia cepacia*, *Penicillium expansum* and *Rhizopus miehei* catalyzed the methanolysis of cooking oil [65,66], but in all of them the reaction takes place with a co-solvent, at higher temperature, and with a percentage (*w*/*w*) of catalyst equal to or greater than the 20% used in this work [67,68,69]. The same lipases, immobilized by hydrophobicity, and the lipase B from *Candida antarctica* Novozym 435 were also tested as catalysts of the methanolysis of oils and fats with variable yields [4,38,70].

## 5. Conclusions

Comparison of six heterogeneous biocatalysts with immobilized OPEr confirmed that both the carrier and the immobilization method strongly impact the efficiency of the enzyme and its suitability for a given bioprocess. Among the soluble enzymes evaluated, only Eversa Transform 2.0 produced biodiesel efficiently in the conditions tested, but the catalytic efficiency improved in all instances after immobilization. The best activity and recyclability in synthesis of biodiesel from oil wastes catalyzed by OPEr were obtained when the protein was immobilized on magnetic nanoparticles by hydrophobicity (which was not accompanied by OPEr hyperactivation), as mCLEAs, or through its oxidized sugar chains (AMNP-CH-OPEr), in which the activity loss in nine cycles was virtually negligible. The efficiency and operational stability of these biocatalysts with immobilized OPEr are comparable to those obtained for free or immobilized Eversa Transform 2.0, showing the biotechnological potential of the non-commercial enzyme. The outstanding enhancement of the stability of the immobilized OPEr contributes to the design of clean and sustainable production of biodiesel and supports its use in other bioprocesses of biotechnological relevance.

## Figures and Tables

**Table 1:** Summary of the immobilization parameters of the biocatalysts with OPEr used in this study.

Nanobiocatalyst	Type of Immobilization	Immobilization Yield (%) ^a^	Specific Activity (mU/mg Carrier) ^b^	Immobilization Efficiency (%) ^c^	Recovered Activity (%) ^d^
**SiMAG-octyl-OPEr**	Hydrophobicity	99 ± 1	851 ± 69	82	80
**AMNP-GA-OPEr**	Covalent	65 ± 8	334 ± 5	69	45
***AG1-GA-OPEr***	Covalent	43 ± 2	405 ± 89	126	41
**AMNP-CH-OPEr**	Covalent	98 ± 3	707 ± 19	57	54
***AG1-CH-OPEr***	Covalent	96 ± 3	896 ± 64	62	58
**mCLEAS-OPEr**	Covalent	99 ± 2	769 ± 58	104	84

^a^ Immobilization yield (%) = (immobilized activity/starting activity) × 100. ^b^ The specific activity of different preparations with versatile lipase attached to magnetic nanoparticles was determined using *p*NPB as the substrate. ^c^ Efficiency (%) = (observed activity/immobilized activity) × 100. ^d^ Recovered activity (%) = (observed activity/starting activity).

**Table 2:** Composition of the mixture obtained after 24 h of reaction of discarded cooking oil and methanol catalyzed by OPEr and its nanobiocatalysts.

Catalyst	% TGs	% DGs	% MGs	% FFAs	% FAMEs
**OPEr**	76.0 ± 4.9	6.2 ± 0.1	0.1 ± 0	2.0 ± 0.3	20.0 ± 0.2
**SiMAG-octyl-OPEr**	4.9 ± 2.9	3.1 ± 1.2	0.2 ± 0.0	14.7 ± 0.9	83.6 ± 2.9
**AMNP-GA-OPEr**	38.1 ± 3.6	9.4 ± 0.5	1.3 ± 0.2	2.2 ± 0.7	50.0 ± 0.8
**AG1-GA-OPEr**	2.7 ± 0.4	4.9 ± 1.4	4.2 ± 2.8	4.5 ± 0.0	84.7 ± 5.0
**AMNP-CH-OPEr**	6.6 ± 1.1	10.2 ± 2.0	0.1 ± 0	3.4 ± 0.3	84.9 ± 4.2
**AG1-CH-OPEr**	3.4 ± 0.1	7.3 ± 0.7	7.0 ± 0.5	8.6 ± 1.6	75.3 ± 9.4
**mCLEAs-OPEr**	4.0 ± 2.1	6.0 ± 4.7	0.1 ± 0.0	2.5 ± 0.1	92.2 ± 5.3

TGs: Triglycerides; DGs: Diglycerides; MGs: Monoglycerides; FFAs: Fatty acids; FAMEs: Methyl esters.
